# ADSCs enhance VEGFR3-mediated lymphangiogenesis via METTL3-mediated VEGF-C m^6^A modification to improve wound healing of diabetic foot ulcers

**DOI:** 10.1186/s10020-021-00406-z

**Published:** 2021-11-13

**Authors:** Jie Zhou, Tianhong Wei, Zhiyou He

**Affiliations:** Department of Burns and Reconstructive Surgery, Xiangya Hospital, Central South University, No. 87 Xiangya Road, Kaifu District, Changsha, 410008 Hunan Province China

**Keywords:** ADSCs, Wound healing, DFU

## Abstract

**Background:**

Adipose-derived mesenchymal stem cells (ADSCs) are an important focus in regenerative medicine. However, the biological function of ADSCs in the wound repair of diabetic foot ulcers (DFUs) remains unclear. This study aimed to determine the underlying mechanisms of ADSCs involved in the wound healing of DFUs.

**Methods:**

The cell surface markers cluster of differentiation 34 (CD34), stromal cell antigen 1 (Stro-1), cluster of differentiation 90 (CD90) and cluster of differentiation 105 (CD105) on ADSCs were identified by flow cytometry. Oil Red O staining and Alizarin Red S staining were performed to identify the multipotential differentiation of ADSCs into adipocytes and bone. The levels of Methyltransferase-like 3 (METTL3), vascular endothelial growth factor C (VEGF-C) and insulin-like growth factor 2 binding protein 2 (IGF2BP2) were assessed by RT-qPCR. CCK-8, Transwell and tubule formation assays were conducted to assess lymphatic endothelial cell (LEC) viability, migration and tubule formation ability, respectively. RIP and RNA pulldown assays were conducted to assess the interaction between IGF2BP2 and VEGF-C. The levels of VEGF-C, VEGFR3, LYVE-1 and IGF2BP2 proteins were assessed by Western blotting. The levels of VEGF-C in LECs were measured by ELISA.

**Results:**

Our findings illustrated that ADSCs accelerate LEC proliferation, migration and lymphangiogenesis via the METTL3 pathway and regulate VEGF-C expression via the METTL3/IGF2BP2-m6A pathway VEGF-C-mediated lymphangiogenesis via the METTL3/IGF2BP2-m6A pathway in DFU mice.

**Conclusion:**

ADSCs enhance VEGFR3-mediated lymphangiogenesis via METTL3-mediated VEGF-C m6A modification to improve wound healing in DFUs, indicating that ADSCs may be regarded as a promising therapeutic strategy to promote wound healing in DFUs.

## Introduction

Diabetic foot ulcers (DFUs) are one of the primary causes of amputation in diabetic patients with diabetic peripheral neuropathy and foot deformities (Amin 2020). Delayed or nonhealing wounds are major complications of DFU (Yin 2020). Therefore, promoting wound repair is the main principle for the treatment of DFU (Cai 2019). Wound repair is a complex and orderly biological process that is mainly divided into the inflammatory phase, granulation proliferation phase, and wound shaping, and a large number of cells, extracellular matrix, cytokines and tissues are involved at each stage of repair (Blumberg et al. 2012).

At present, the potential role of stem cells in wound repair treatment has been confirmed (Foroutan et al. 2018). Adipose-derived mesenchymal stem cells (ADSCs), which are a type of adult mesenchymal stem cell that exists in adipose tissue, have the potential for self-renewal and multidirectional differentiation (Huang et al. 2019). Increasing research indicates that ADSCs play a pivotal role in the process of wound repair. For example, Aurora A kinase (AURKA) promotes diabetic wound repair by enhancing ADSC autophagy (Yin et al. 2020). Activin B regulates ADSC migration by activating the MAPK pathway, resulting in skin wound healing (Zhang et al. 2017). In addition, ADSCs shorten wound healing time and prevent scar formation in skin-deficient mice by activating the PI3K/Akt pathway (Zhang et al. 2018). Shu et al. showed that the combination of ADSCs and exendin-4 effectively promotes diabetic wound healing (Seo et al. 2017). Li et al. showed that ADSCs accelerate skin wound healing by promoting vascularization in DFU rats (Li et al. 2018). Furthermore, ADSCs have the ability to mediate DFU wound healing through mitochondrial transfer and the secretion of p-hydroxybenzoic factor and exosomes (Li et al. 2018). However, the specific mechanism by which ADSCs promote wound healing in DFUs has not been fully elucidated. Furthermore, studies have shown that ADSCs promote lymphangiogenesis by secreting lymphangiogenic factors in vitro (Ahmadzadeh, et al. 2020). In addition, multiple growth factors secreted by ADSCs have also attracted extensive attention (Geng et al. 2019). However, the role and underlying mechanisms of ADSCs involved in the wound repair of DFUs remain unclear.

VEGFRs belong to the family of receptor tyrosine kinases (RTKs), which play a central role in endothelial functions, including cell proliferation and survival, angiogenesis and lymphangiogenesis (Ma et al. 2019). As a vital cytokine in the process of lymphangiogenesis, vascular endothelial growth factor C (VEGF-C) plays an important role in the process of various diseases. Xie et al. reported that the overexpression of VEGF-C in ADSCs reduces skin damage by ultraviolet rays (Xie et al. 2019), indicating that ADSCs may accelerate skin wound healing by enriching VEGF-C. Moreover, increasing evidence shows that ADSCs are a pivotal factor in the process of lymphangiogenesis and that the promotion of lymphangiogenesis is essential for tissue regeneration (Krawczenko et al. 2020), Studies have shown that VEGFC-induced downregulation of vascular endothelial growth factor receptor 3 (VEGFR3) is involved in lysosomal degradation and that Ephrin-B2 controls VEGF-induced angiogenesis and lymphangiogenesis via the AKT/ERK pathway (Smith et al. 2010). Thus, we speculate that ADSCs may play a vital role in VEGFR3-mediated lymphangiogenesis. Methyltransferase-like 3 (METTL3) is an RNA methyltransferase implicated in mRNA biogenesis, decay, and translation control via N6-methyladenosine (m6A) modification (Lin et al. 2016). m6A modification is the most common internal posttranscriptional modification of mammalian mRNA and mainly regulates the levels of mRNA molecules by affecting the stability of mRNA, nuclear movement and translation initiation (Yu et al. 2018). METTL3 knockdown inhibits the levels of VEGF-C (Wang et al. 2018). However, whether METTL3 regulates VEGF-C m6A modification remains unclear.

Insulin-like growth factor 2 binding proteins (IGF2BPs), including IGF2BP1/2/3, are a distinct family of m6A readers that target thousands of mRNA transcripts by recognizing the consensus GG (m6A) C sequence (Huang et al. 2018). IGF2BPs promote the stability and storage of their target mRNAs, such as MYC, in an m6A-dependent manner under normal and stress conditions and thus affect gene expression output (Samuels et al. 2020). Our preliminary findings show that IGF2BP2 binds to VEGF-C mRNA. Therefore, we speculate that METTL3 may increase the level of VEGF-C m6A, enhance the binding of IGF2BP2 and VEGF-C, prevent the degradation of VEGF-C mRNA, and promote VEGF-C-induced VEGFR3-mediated lymphangiogenesis (Alishekevitz et al. 2016).

In summary, in this paper, we aimed to investigate whether ADSCs enhance VEGFR3-mediated lymphangiogenesis via METTL3-mediated VEGF-C m6A modification to improve wound healing in DFUs. Our findings may provide a theoretical basis for the clinical application of ADSCs in wound repair in DFUs.

## Materials and methods

### Animals

C57BL/6 mice used in this study were purchased from SJA Laboratory Animal Co., Ltd. (Hunan, China). During the feeding process, all animals were kept in a cage (n = 6), and sufficient water and feed were given every day, along with 12 h of light per day. The animal experiments in this paper were conducted in accordance with the Guidelines for the Care and Use of Experimental Animals published by the National Institutes of Health (NIH). All animal behaviours received approval from the Animal Care and Use Committee of Xiangya Hospital Central South University.

### ADSC isolation and identification

ADSCs were obtained from adipocytes by limiting dilution cloning culture, and adipocytes were segregated from the inguinal subcutaneous adipose tissue of healthy C57BL/6 mice 4 days after birth by collagenase digestion. ADSCs belongs to auto graft cells. High glucose Dulbecco's modified Eagle's medium (DMEM, Roche, Basel, Switzerland), which was supplemented with 1% penicillin–streptomycin solution (Solarbio, Beijing, China) and 10% FBS, was used to culture ADSCs, and the cells were cultured in a humid incubator containing 5% CO_2_ at 37 ℃. All cells were cultured under high glucose (30 mM, glucose) conditions (Li et al. 2018). When the ADSCs grew to 80–90% confluence, the ADSCs were digested with trypsin, and the cells were filtered with a cell strainer with a pore size of 100 µm. Subsequently, ADSCs were resuspended in precooled 1 × PBS buffer at a density of 2 × 105 cells/mL. The ADSCs were stained with anti-Cluster of differentiation 34 (CD34) (ab81289, 1:1000), anti-stromal cell antigen 1 (Stro-1) (ab214086, 1:1000), anti-cluster of differentiation 90 (CD90) (ab133350, 1:1000) and anti-cluster of differentiation 105 (CD105) (ab252345, 1:1000) monoclonal antibodies labelled with phycoglobin (PE) or luciferin isothiocyanate (FITC). All antibodies were purchased from Abcam (Cambridge, UK). Next, after washing, the ADSCs were stained with FITC-labelled secondary antibody for 30 min and washed twice with 1 × PBS. Finally, data were collected using flow cytometry (FACSCalibur) and analysed using CellQuest6.0 software (both from BD Biosciences).

### Oil Red O and alizarin Red S staining

The osteogenic differentiation and lipid differentiation of ADSCs were observed by Alizarin Red S and Oil Red O staining, respectively. After the 3rd passaging, ADSCs were digested with 0.25% trypsin. A cell concentration of 0.5 × 10^5^ cells/mL was used, and the cells were inoculated into a 6-well plate. When the cell confluences reached 90%, the induction medium was replaced. A concomitant induction medium (0.5 mM iso-butyl methyl xanthine (IBMX), dexamethasone, 1 mM to 10 mM insulin, 200 mM indomethacin, 1% antibiotic/antifungal drugs) and osteogenesis induction medium (0.01 mM 2 hydroxy vitamin D3, 1, 25–50 mM-2-phosphoric acid, ascorbic acid 10 mM b-glycerophosphate, 1% antibiotic/antifungal medicine) from Sigma-Aldrich (St Louis, MO, USA) were used for osteogenesis induction. Oil red O staining was performed 2 weeks after osteogenesis induction, and alizarin red S staining was performed 4 weeks after osteogenesis induction.

### A full thicknes wound model

To evaluate the therapeutic effects of METTL3- and IGF2BP2-downregulated ADSCs on diabetic wound closure in vivo, a mouse model was established. Briefly, following fur removal from the dorsal surface, the mice were anaesthetized with sodium pentobarbital (0.5 mg/g), and after 72 h treatment, a 10-mm full-thickness round excisional skin wound was made on the back of each mouse with a sterile 10-mm punch biopsy tool (Xu et al. 2020).Thirty-six male C57BL/6 mice were randomly divided into 6 groups, during the feeding process, all animals were kept in a cage (n = 6), and sufficient water and feed were given every day, along with 12 h of light per day: normal foot ulcer group, DFU group, ADSC DFU group, ADSC-sh-NC DFU group, ADSC-sh-METTL3 DFU group and ADSC-sh-IGF2BP2 DUF group, with 6 mice in each group. In the normal foot ulcer group, the mice were untreated. Diabetes was induced by intraperitoneal (i.p.) injection of 150 mg/kg body weight streptozotocin (STZ). Mice with blood glucose levels ≥ 16.7 mmol/L, and mice with non-fasting blood glucose levels > 300 mg/dL were considered diabetic. In the diabetic foot ulcer group, the diabetic mice were untreated. In the ADSC group, the diabetic mice received approximately 2.5 × 10^5^ ADSCs in phosphate-buffered saline (PBS) injected intradermally daily around the wound tissue. In the ADSC-sh-NC group, the diabetic mice received approximately 2.5 × 10^5^ sh-NC-transfected ADSCs in PBS injected intradermally daily around the wound tissue. In the ADSC-sh-METTL3 group, the diabetic mice received sh-METTL3-transfected ADSCs in PBS injected intradermally daily around the wound tissue. In the ADSC-sh-IGF2BP2 group, the diabetic mice received sh-IGF2BP2-transfected ADSCs in PBS injected intradermally daily around the wound tissue. After 28 days treatment, all mice were sacrificed.

### Primary lymphatic endothelial cells (LECs) isolation

A small piece of subcutaneous tissue (approximately 0.3 cm^2^) was cut off from the back of both hind feet on the wound surface of anaesthetized DFU model mice. The tissue was chopped with a sterile razor, and then the chopped tissue was placed in an Eppendorf tube containing 200 μL TCH buffer (1 mg/mL collagenase V, 0.125% trypsin–EDTA and 0.3 mg/mL hyaluronidase) (Thermo Fisher Scientific, Waltham, Massachusetts, USA). The test tube was rotated upside down at 37 °C for 15 min. The chunks were allowed to precipitate. The supernatant was removed and transferred to a new Eppendorf tube containing 500 μL HAM F12 and 20% foetal bovine serum (FCS) (Solarbio, Beijing, China) to inactivate the enzyme. A further 200 μL TCH buffer was added to the first test tube containing tissue fragments, which was then rotated for another 15 min at 37 °C. These two operations were repeated to combine the supernatant and the remaining tissue fragments, and then the precipitates were resuspended in HAM F12 and 20% FCS, centrifuged at a speed of 1000 rpm for 4 min, and placed in a 3.5 cm petri dish. The culture was refed regularly with fresh medium, and a layer of confluent cells was prepared for subculture within a week.

### Cell treatment and transfection

For cell transfection, sh-METTL3, sh-IGF2BP2, pcDNA-VEGF-C and pcDNA-METTL3 plasmids were synthesized by Sangon Biotech (Shanghai, China), and the negative controls were sh-NC and pcDNA-NC. According to the manufacturer's instructions, transfection was carried out using Lipofectamine™ 3000 Transfection Reagent (Takara, Kusatsu, Japan). Forty-eight hours after transfection, ADSCs were used in the follow-up experiment.

### RT-qPCR

TRIzol reagent (Invitrogen, CA, USA) was used according to the manufacturer's instructions to extract the total RNA from tissue samples or fibroblasts. An M-MLV Reverse Transcriptase (RNase H) kit (Takara, Kusatsu, Japan) was used to synthesize cDNA. RT-qPCR was performed as previously described (Chen et al. 2018). The primers used in this study are shown in Table 1.

**Table 1:** Primers used in this paper

Primer name	Primer sequences
F- METTL3	5′-TGACTCCAGTGCTGATCGAC-3′
R- METTL3	5′-CTGCGCATCTCATCATCTGT-3′
F-IGF2BP2	5′-AGAGGCCTTTGAGAACGACA-3′
R-IGF2BP2	5′-TGGAGAAGTATCCGGAGTGG-3′
F-VEGF-C	5′-GAAATCAGCCCCTGAATCCT-3′
R-VEGF-C	5′-CACAGCGGCATACTTCTTCA-3′
F-U6	5′-CTCGCTTCGGCAGCACA-3′
R-U6	5′-AACGCTTCACGAATTTGCGT-3′
F-GAPDH	5′-AGCCCAAGATGCCCTTCAGT-3′
R-GAPDH	5′-CCGTGTTCCTACCCCCAATG-3′

### Western blot

Total proteins were isolated from mouse wound tissue or ADSCs and then transfected with corresponding plasmids and reagents using cell lysis buffer (Semel Fisher Technology Co., Shanghai, China). Western blotting was performed as described previously (Su et al. 2018). Antibodies against VEGF-C (sc-374628), IGF2BP2 (sc-271785), VEGFR3 (sc-514825), LYVE-1 (sc-65647) and GAPDH (sc-365062) were purchased from Santa Cruz (Santa Cruz, Shanghai, China, 1:1000). Horseradish peroxidase (HRP)-coupled goat anti-rabbit IgG secondary antibody (sc-2004) was used as the secondary antibody (Santa Cruz, Shanghai, China, 1:2000). The optical density quantification of protein bands was evaluated by ImageJ software (ImageJ Software, Inc.).

### ADSC-conditioned medium

When 80% confluence was reached, ADSCs were incubated with 10 mL endothelial cell base medium (ECBM, PromoCell GmbH) containing 0.5% FBS (foetal bovine serum, FBS excellent; Biochrom AG) for 48 h. Then, ADSC-conditioned medium (ADSC-CM) was collected for further study, according to previous reports (Wang et al. 2018). For use in the functional research, ADSC-CM was further concentrated 10 times in 30 min by using an ultrafiltration membrane with a molecular weight cut-off of 3 kDa (Millipore, Billerica, MA, USA). In addition, ADSC-CM with different dilutions were prepared by diluting ECBM containing 0.5% FBS.

### Tube formation assay

A tubule formation assay was performed to evaluate the ability of LECs to form capillaries. First, the μ-Slide angiogenic plate (ibidi GmbH, Munich, Germany) coated with 10 μL/well of growth factor reduced Matrigel® (Corning Inc.) (Munich, Germany) was incubated at 37 °C and 5% CO_2_ for 30 min to induce polymerization. Then, before starting the experiment, the cells were starved overnight in ECBM containing 0.5% FBS. LECs were inoculated at a density of 8 × 10^3^ cells per well and suspended in 50 μL LADSC-CM. Finally, after incubation for 16 h, the number of rings in each well and the length of the main tubule were quantified. The digital image was analyzed by using a Wimasis image analyser (Onimagin Technologies SCA, Cordoba, Spain).

### ELISA

One millilitre of protein extraction reagent (Beyotime, Nanjing, China) was used to lyse the fibroblasts, and the levels of VEGF-C in the supernatant of LECs were determined using an ELISA kit (Biomatik, Ca, EKU08098, sensitivity: 6.1 pg/mL, range: 15.6–1000 pg/mL) according to the manufacturer's instructions.

### Proliferation assay

The viability of the LECs was analysed by using Cell Counting Kit-8 (Beyotime, Nanjing, China) according to the manufacturer's instructions. In detail, LECs were inoculated into a 96-well plate at a density of 3 × 10^3^ cells per well and cultured in ECGM. After 4 h of culture, nonadherent cells were removed by washing with PBS. The cells were then stimulated with 500 μL ADSC-CM for 24 h, and a total of 10 μL CCK-8 solution was added to each well at a specified time. Finally, the medium mixed with CCK-8 solution was added to the new 96-well plate. A fluorescence microplate reader was used to detect the absorbance at 450 nm, which reflected the cell viability.

### Migration assay

A Transwell chamber with a membrane pore size 8 μm in diameter was used to evaluate cell migration. In detail, LECs were inoculated into a 12-well plate at a density of 1 × 10^5^ cells per well and cultured in ECGM. After 4 h of culture, nonadherent cells were removed by washing with PBS. Then, LECs stimulated with 1 mL ADSC-CM for 24 h were inoculated into the upper chamber in serum-free medium. At the same time, medium containing 20% FBS was added to the bottom chamber. After incubation for 24 h, the remaining cells on the upper side of the membrane were removed with cotton swabs, and the cells at the bottom of the membrane were fixed with 4% paraformaldehyde and stained with 0.1% crystal violet. The stained cells were quantified in five randomly selected areas under an inverted microscope, and the average value was used to determine the invasive ability of the cells.

### MeRIP assay

MeRIP analysis was performed as described previously, with minor modifications (Li et al. 2019).

In detail, 50 μg of total RNA was extracted and purified to deplete ribosomal RNA from total RNA using a RiboMinus eukaryotic kit v2. Next, the RNA was cut into fragments of approximately 100 nt using an RNA fragmentation reagent (AM8740, Invitrogen, CA, USA). Approximately 1/10 of the RNA was saved at RiboBio (Guangzhou, China) as an input control for further RNA sequencing. The remaining RNA was incubated with an anti-M6A antibody (202, 203 Synaptic Systems) at 4 °C for 1 h and then mixed overnight with prewashed Pierce Protein A/G Magnetic Beads (88, 803) in immunoprecipitation buffer at 4 °C. The m6A antibody was digested with protease K digestion buffer, and methylated RNA was purified by RiboBio (Guangzhou, China) for further MeRIP sequencing.

### RIP assay

According to the manufacturer's instructions, an RNA immunoprecipitation kit (Thermo Scientific, Shanghai, China) was used for RIP analysis. In short, magnetic beads coated with 5 μg specific antibodies against mouse immunoglobulin G (Invitrogen, CA, USA) or IGF2BP2 (Santa Cruz, Shanghai, China, 1RU 500) were incubated overnight with the prepared cell lysate at 4 °C. Subsequently, the RNA–protein complex was washed 6 times and incubated with protease K digestion buffer. Finally, RNA was extracted by the phenol–chloroform RNA extraction method. The relative interaction between IGF2BP2 and VEGF-C transcripts was determined by RT-qPCR and normalized according to the input.

### RNA pulldown

RNA was first transcribed by a MEGAscript T7 transcription kit (Thermo Scientific, (Shanghai, China). Then, by using a Pierce RNA 3' terminal desulfurization biotinylating kit (Thermo Scientific, Shanghai, China), the amplified RNA was labelled with desulfurized biotin. Finally, the Pierce magnetic RNA protein pulldown kit (Thermo Scientific, Shanghai China) was used for the RNA pulldown assay. A maximum of 50 pmol biotinylated RNA was mixed with 2 mg protein lysate and 50 μL magnetic streptavidin beads. After incubation and washing three times, the streptavidin beads were boiled and used for immunoblotting.

### Statistical analysis

The mean ± SD represents data from three independent experiments. All the data were statistically analysed using GraphPad Prism version 6.0 software (GraphPad Software, Inc.). Student’s *t*-test was used to compare the two groups, and one-way ANOVA followed by Tukey’s post-hoc test was used to compare multiple groups. The difference was deemed statistically significant when *P* < 0.05.

## Results

### ADSCs isolation, culture and identification

**Fig. 1:**
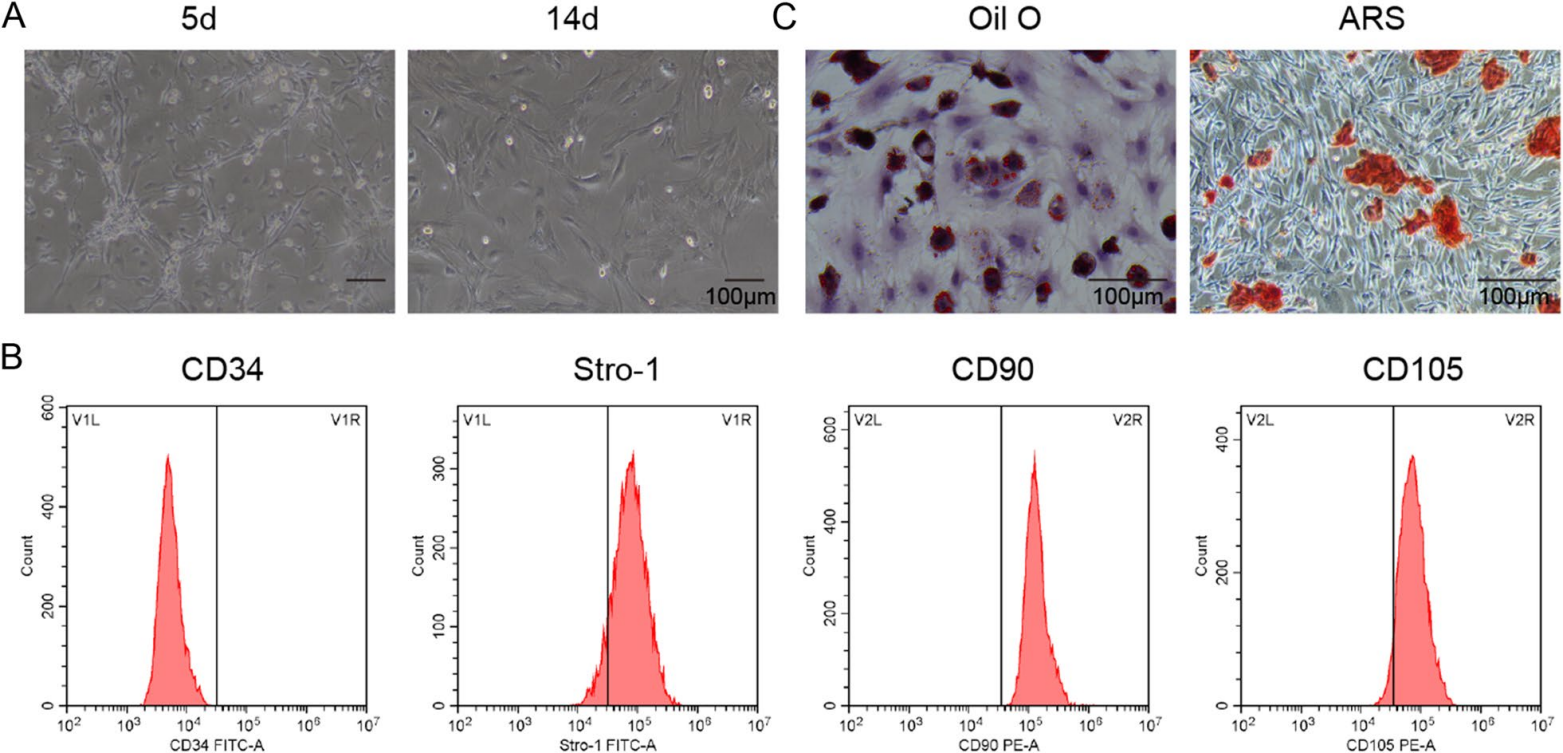
The isolation, culture and identification of ADSCs. **A** An inverted microscope was used to observe the morphology of ADSCs (scale bar: 100 μm). **B** Flow cytometry was carried out to assess the surface marker antigens on ADSCs. **C** Differentiation potentials of ADSCs were assessed by Alizarin Red S staining and Oil Red O staining (scale bar: 100 μm)

An inverted microscope was used to observe the morphology of ADSCs, which were isolated from the inguinal subcutaneous adipose tissue of mice. The results indicated that MSCs showed a fusiform shape and began adherent growth following 24 h of culture (Fig. 1A, left). The majority of cells showed fibroblast-like morphology and an increase in volume after culturing for 6 days (Fig. 1A, right). Furthermore, flow cytometry and staining with Oil Red O and Alizarin Red S were performed to identify the characteristics of the ADSCs. The results of flow cytometry analysis showed that the ADSCs were positive for the mesenchymal markers CD90, Stro-1 and CD105 and negative for the haematopoietic marker CD34 (Fig. 1B). This is consistent with the surface antigen characteristics of ADSCs. In addition, as shown in Fig. 1C, many red lipid droplets were visible by Oil Red O staining after adipogenesis induction, and black calcium nodules appeared by Alizarin Red S staining after osteogenesis induction.

### ADSCs accelerates LEC proliferation, migration and lymphangiogenesis via METTL3 pathway

**Fig. 2:**
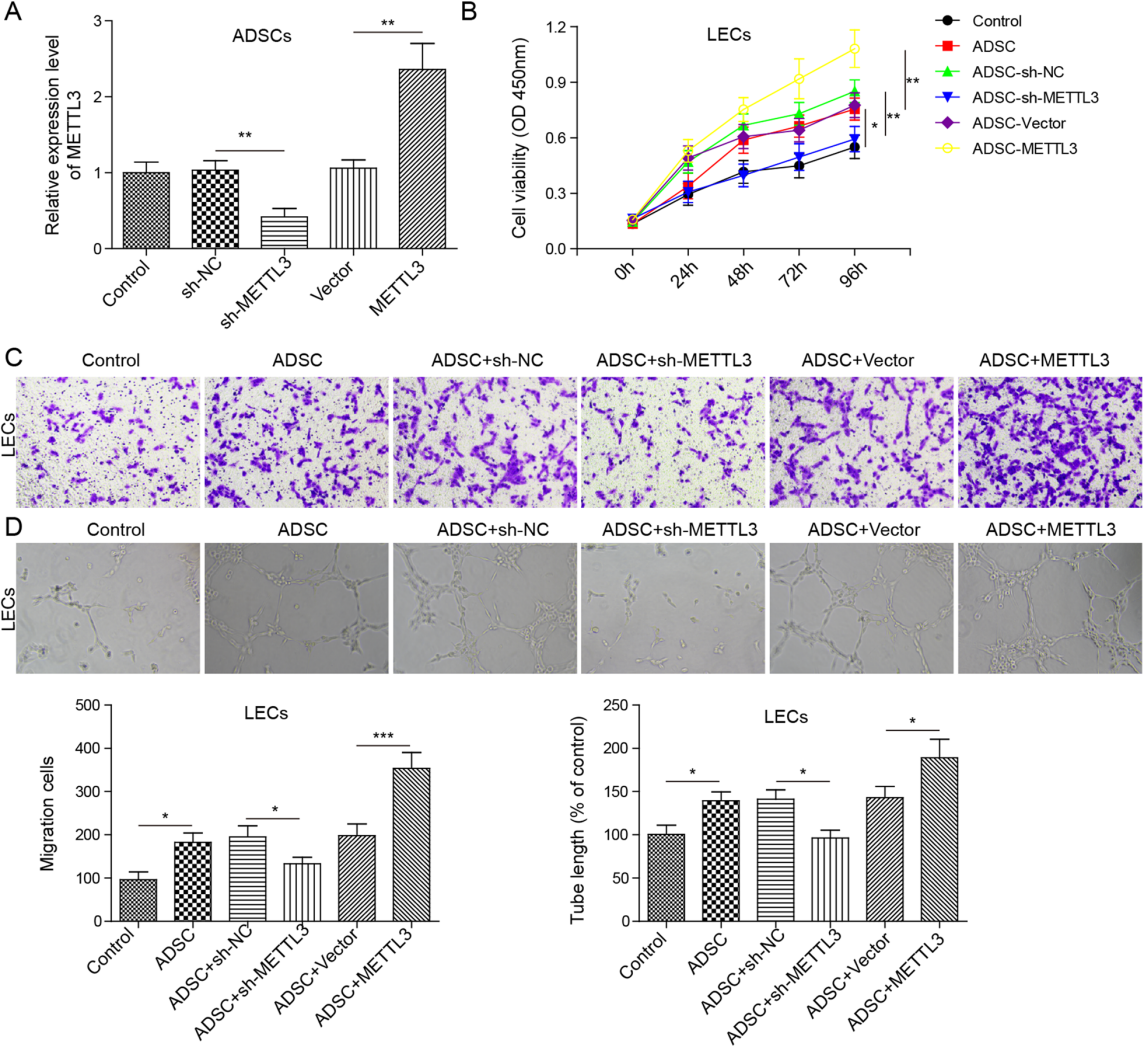
ADSCs accelerate LEC proliferation, migration and lymphangiogenesis via the METTL3 pathway. sh-METTL3 and pcDNA-METTL3 plasmids were transfected into ADSCs for METTL3 knockdown and overexpression, respectively. sh-NC served as the negative control for sh-METTL3 plasmids, and pcDNA-empty vector served as the negative control for pcDNA-METTL3 plasmids. Subsequently, LECs were stimulated with ADSC-CM for 24 h. **A** RT-qPCR was performed to assess the levels of METTL3. **B** CCK-8 was conducted to assess LEC viability. **C** Transwell assays were carried out to evaluate LEC migration. **D** A tubule formation assay was performed to assess the tubule formation ability of LECs. Data represent the mean of three independent experiments. Error bars represent SD. *P < 0.05, **P < 0.01 or ***P < 0.001

To investigate the underlying mechanism of ADSCs in the wound healing of DFUs, first, the effect of ADSCs on LEC bioactivities and lymphangiogenesis was analysed. RT-qPCR analysis showed that the transfection of sh-METTL3 significantly inhibited METTL3 expression in ADSCs compared with the control. However, the results of the transfection with pcDNA-METTL3 plasmids showed the reverse trend (*P* < 0.01, Fig. 2A). CCK-8, cell migration and tubule formation assays indicated that compared with the control, ADSCs accelerated the viability, migration and tubule formation of LECs. Moreover, compared with the negative control (NC), METTL3-knockdown ADSCs inhibited the viability, migration and tubule formation of LECs, whereas METTL3-overexpressing ADSCs showed the opposite effect. Namely, METTL3-overexpressing ADSCs accelerated the viability, migration and tubule formation of LECs (*P* < 0.05, *P* < 0.01 or *P* < 0.001, Fig. 2B–D). These findings revealed that ADSCs accelerate LEC proliferation, migration and lymphangiogenesis via METTL3.

### ADSCs regulate VEGF-C expression via METTL3/IGF2BP2-m^6^A pathway

**Fig. 3:**
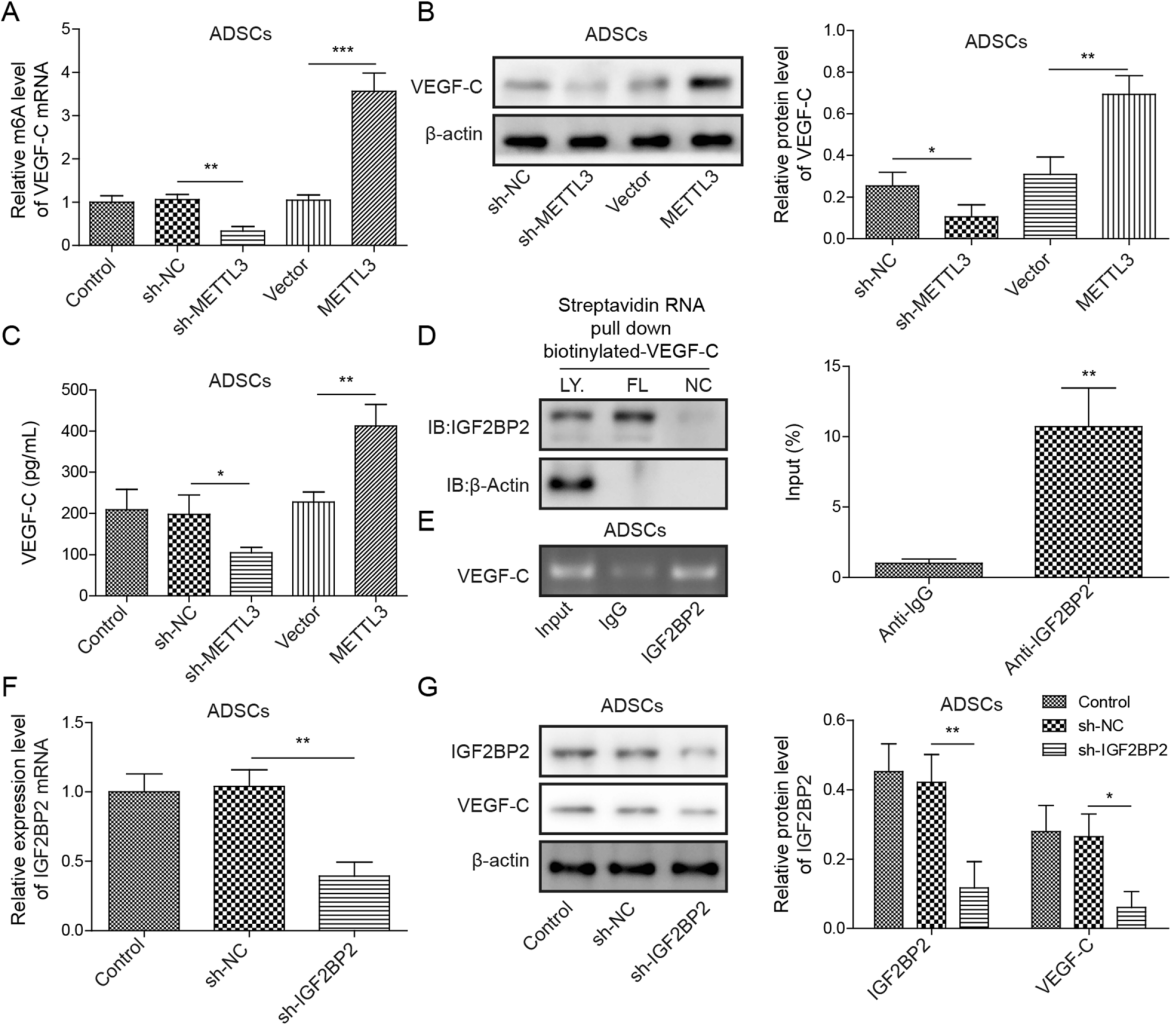
ADSCs regulate VEGF-C expression via the METTL3/IGF2BP2-m6A pathway. sh-METTL3 and pcDNA-METTL3 plasmids were transfected into ADSCs for METTL3 knockdown and overexpression, respectively. sh-NC served as the negative control for sh-METTL3, and the pcDNA-empty vector served as the negative control for pcDNA-METTL3 plasmids. **A** MeRIP-qPCR was performed to assess the m^6^A levels of VEGF-C mRNA. **B** Western blotting was conducted to measure the levels of VEGF-C proteins. **C** An ELISA was carried out to assess the levels of VEGF-C in the supernatant of ADSCs. **D**, **E** RNA pulldown and RIP assays were performed to evaluate the interaction between VEGF-C and IGF2BP2. Immunoblotting of IGF2BP2 after RNA pulldown assay with cell lysate (Ly) and full-length biotinylated VEGF-C (FL); NC indicates the beads. sh-IGF2BP2 plasmids were transfected into ADSCs for IGF2BP2 knockdown, and sh-NC served as the negative control for sh-IGF2BP2 plasmids. **F** RT-qPCR was performed to assess the levels of IGF2BP2. **G** Western blotting was conducted to measure the protein levels of IGF2BP2 and VEGF-C. Data represent the mean of three independent experiments. Error bars represent SD. *P < 0.05, **P < 0.01 or ***P < 0.001

To investigate the effect of ADSCs on VEGF-C expression, the potential molecular mechanism was investigated. RT-qPCR analysis showed that compared with the control, the m6A levels of VEGF-C in ADSCs were inhibited by METTL3 knockdown but accelerated by METTL3 overexpression (*P* < 0.01 or *P* < 0.001, Fig. 3A). These findings indicate that METTL3 accelerates the m6A levels of VEGF-C in ADSCs. Subsequently, Western blotting and ELISA analysis indicated that compared with the control, the levels of VEGF-C in ADSCs were inhibited by METTL3 knockdown, whereas METTL3 overexpression had the opposite effect (*P* < 0.05 or *P* < 0.01, Fig. 3B and C), indicating that METTL3 accelerates the levels of VEGF-C in ADSCs. Furthermore, an RNA pulldown assay verified that IGF2BP2 bound the VEGF-C full-length transcripts (*P* < 0.01, Fig. 3D). RIP assay analysis indicated that there was an interaction between IGF2BP2 and VEGF-C (*P* < 0.01, Fig. 3E). Moreover, RT-qPCR and Western blot analysis showed that compared with the control, IGF2BP2 knockdown inhibited VEGF-C expression in ADSCs (*P* < 0.05 or *P* < 0.01, Fig. 3F and G), indicating that IGF2BP2 accelerates VEGF-C expression in ADSCs. Based on the above results, we suggest that ADSCs may regulate VEGF-C expression via the METTL3/IGF2BP2-m6A pathway.

### ADSCs regulate VEGF-C-mediated lymphangiogenesis via METTL3/IGF2BP2-m^6^A pathway

**Fig. 4:**
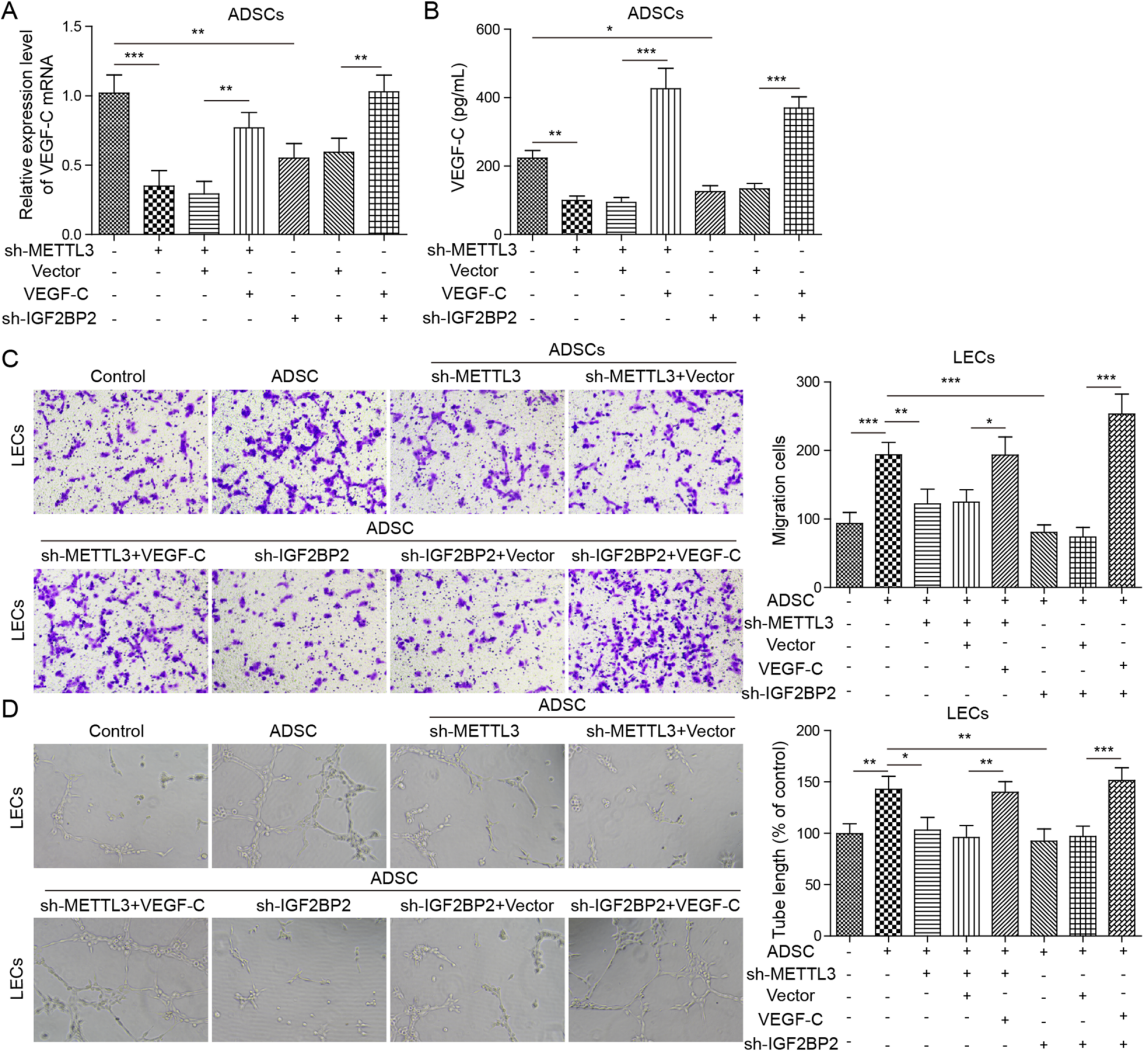
ADSCs regulate VEGF-C-mediated lymphangiogenesis via the METTL3/IGF2BP2-m^6^A pathway. sh-METTL3 or sh-IGF2BP2 and pcDNA-VEGF-C were cotransfected into ADSCs. sh-NC served as the negative control for sh-METTL3 or sh-IGF2BP2, and the pcDNA-empty vector served as the negative control for pcDNA-VEGF-C plasmids. Subsequently, transfected ADSCs were cocultured with LECs. **A** RT-qPCR was performed to assess the levels of VEGF-C. **B** An ELISA was conducted to assess the levels of VEGF-C. **C** Transwell assays were carried out to evaluate LEC migration. **D** A tubule formation assay was performed to assess the tubule formation ability of LECs. Data represent the mean of three independent experiments. Error bars represent SD. *P < 0.05, **P < 0.01 or ***P < 0.001

To further investigate the underlying mechanisms of ADSCs in VEGF-C-mediated lymphangiogenesis, sh-METTL3 or sh-IGF2BP2 plasmids and pcDNA-VEGF-C were cotransfected into ADSCs. sh-NC served as the negative control for sh-METTL3 or sh-IGF2BP2 plasmids, and pcDNA-empty vector served as the negative control for pcDNA-VEGF-C plasmids. Subsequently, LECs were stimulated with ADSC-CM for 24 h. RT-qPCR and ELISA analysis showed that compared with the control, the VEGF-C mRNA levels in ADSCs were inhibited by METTL3 knockdown but alleviated by VEGF-C overexpression. Consistently, compared with the control, IGF2BP2 knockdown inhibited the levels of VEGF-C mRNA in ADSCs, and VEGF-C overexpression alleviated the inhibitory effect (*P* < 0.05, *P* < 0.01 or *P* < 0.001, Fig. 4A and B). Moreover, Transwell and tubule formation assays indicated that compared with the control, the migration and tubule formation ability of LECs were increased by ADSCs. METTL3 knockdown-ADSCs and IGF2BP2 knockdown-ADSCs both inhibited the migration and tubule formation ability of LECs, and the inhibitory effect was alleviated by VEGF-C overexpression (*P* < 0.05, *P* < 0.01 or *P* < 0.001, Fig. 4C and D). These findings revealed that ADSCs regulate VEGF-C-mediated lymphangiogenesis via the METTL3/IGF2BP2-m^6^A pathway.

### ADSCs regulate VEGF-C-mediated lymphangiogenesis via METTL3/IGF2BP2-m^6^A pathway in DFU mice

**Fig. 5:**
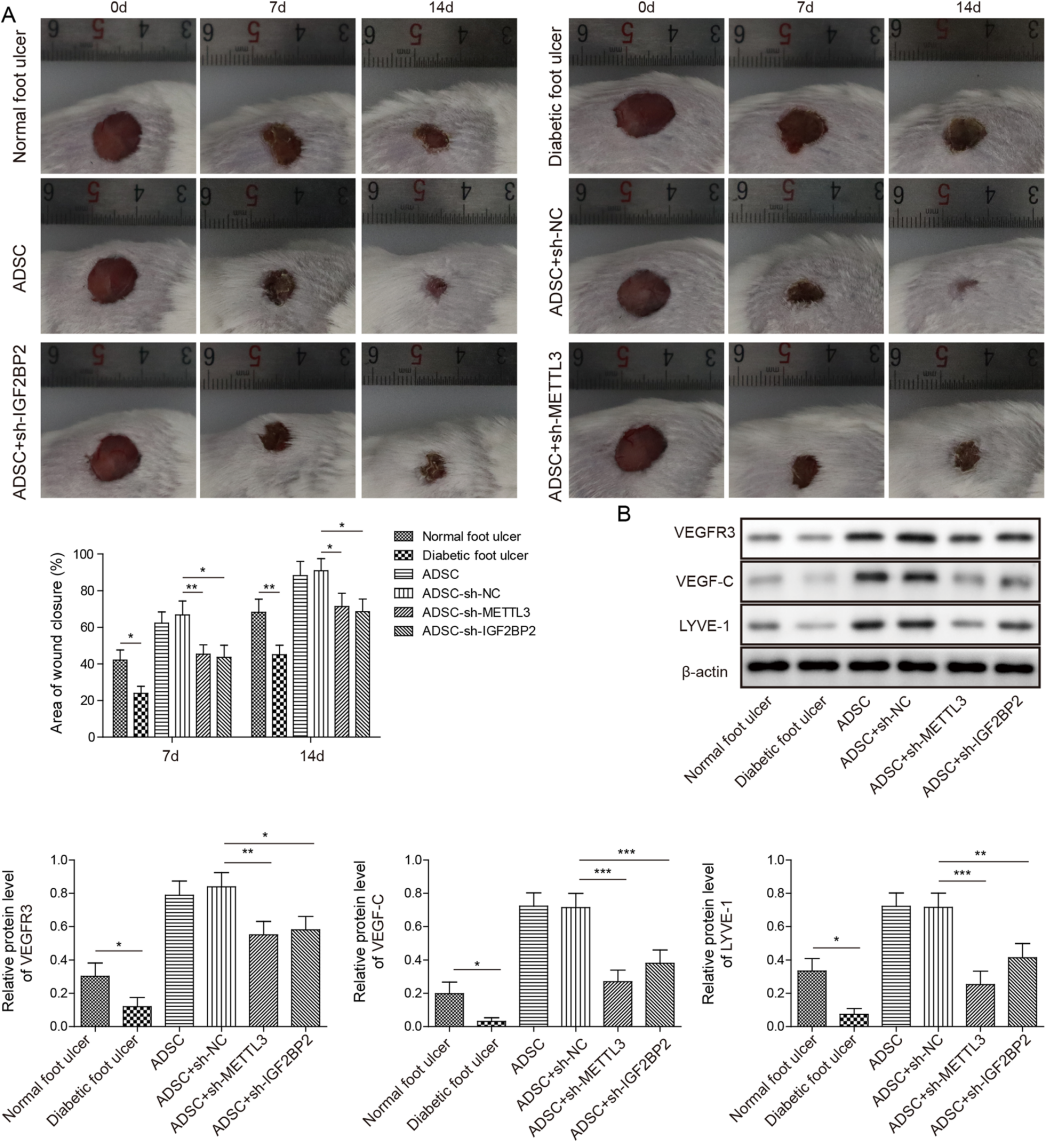
ADSCs regulate VEGF-C-mediated lymphangiogenesis and macrophage infiltration via the METTL3/IGF2BP2-m^6^A pathway in DFU mice. Sh-METTL3 or sh-IGF2BP2 plasmids were transfected into ADSCs for METTL3 or IGF2BP2 knockdown. sh-NC served as the negative control for sh-METTL3 or sh-IGF2BP2. Subsequently, untreated ADSCs or transfected ADSCs were injected into DFU mice. (N = 5) **A** The wound closure statistics of DFU mice. **B** Western blotting was conducted to detect the protein levels of VEGFR3, VEGF-C and LYVE-1. Data represent the mean of five independent experiments. Error bars represent SD. *P < 0.05, **P < 0.01 or ***P < 0.001

To further explore the molecular mechanism by which ADSCs promote wound healing in mice, sh-METTL3 or sh-IGF2BP2 plasmids were transfected into ADSCs for METTL3 or IGF2BP2 knockdown, respectively. sh-NC served as the negative control for sh-METTL3 or sh-IGF2BP2 plasmids. To evaluate the therapeutic effects of METTL3- or IGF2BP2-knockdown ADSCs on diabetic wound closure, a mouse model was established according to previous reports. Subsequently, untreated ADSCs or transfected ADSCs were injected into the DFUs of C57BL/6 mice for 4 weeks. Wound closure statistics showed that the wound healing rate of DFU mice was lower than that in the normal foot ulcer group, whereas ADSCs accelerated the wound healing rate of DFU mice. In addition, compared with the negative control, both METTL3 knockdown- and IGF2BP2 knockdown ADSCs arrested the wound healing rate of DFU mice (*P* < 0.05 or *P* < 0.01, Fig. 5A), indicating that ADSCs may regulate wound healing in DFU mice via the METTL3/IGF2BP2 pathway. LYVE-1 is a marker of lymphangiogenesis. Western blot analysis showed that the protein levels of VEGFR3, VEGF-C and LYVE-1 in DFU mice were significantly lower than those in normal foot ulcers of mice, whereas ADSCs accelerated their levels in DFU mice. Furthermore, compared with the negative control, the protein levels of VEGFR3, VEGF-C and LYVE-1 in DFU mice were significantly inhibited by METTL3 knockdown- and IGF2BP2 knockdown ADSCs (*P* < 0.05, *P* < 0.01 or *P* < 0.001, Fig. 5B). These findings revealed that ADSCs regulate VEGF-C-mediated lymphangiogenesis via the METTL3/IGF2BP2-m^6^A pathway in DFU mice.

## Discussion

DFU is one of the most serious complications and common causes of delayed wound healing (Li et al. 2020). ADSCs have attracted widespread attention due to their ability to secrete multiple types of cytokines (Huang et al. 2019). In recent years, increasing evidence has indicated that ADSCs play a pivotal role in wound repair because of their secretion of many natural growth factors (Badhe and Nipate 2019). For example, research by Sun et al. shows that miR-590–3-5p regulates ADSCs to regulate the pararenal secretion of VEGFA to promote DFU wound healing (Sun et al. 2020). We aimed to illustrate the specific mechanism by which ADSCs improve wound healing in DFUs. In this paper, our findings demonstrated that ADSCs enhance VEGFR3-mediated lymphangiogenesis via METTL3-mediated VEGF-C m6A modification to improve wound healing of diabetic foot ulcers, indicating that ADSCs can be applied for the clinical treatment of DFU.

It has been reported that ADSCs promote lymphangiogenesis by promoting LEC proliferation and migration (Wang et al. 2018; Huang et al. 2019). Studies have shown that ADSCs are widely involved in the regulation of wound healing. For example, ADSCs play an important role in accelerating wound repair in DFU mice by activating the PI3K/Akt signalling pathway (Zhang et al. 2018). ADSCs promote the migration of human skin fibroblasts and wound repair by promoting the expression of ColA1 and MMP-1(Cho et al. 2010). In addition, studies have shown that the wound repair function of ADSCs is due to their secreted cytokines, including angiogenesis and lymphangiogenesis factors (Ahmadzadeh et al. 2020; Chen 2020). Our findings showed that METTL3 knockdown-ADSCs inhibits cell viability, migration and tubule information ability of LEC, while METTL3 overexpressed-ADSCs has the reverse trend. These findings indicate that ADSCs accelerates LEC proliferation, migration and lymphangiogenesis via METTL3 pathway.

In mammalian cells, the modification of m6A is catalysed by a methyltransferase complex that contains METTL3, METTL14 and WTAP (WT1-associated protein). By affecting the m6A level of mRNA, METTL3 changes the recognition ability of m6A readers to mRNA molecules, thus affecting the stability of mRNA (Song et al. 2019). In our study, the results showed that METTL3 knockdown-ADSCs inhibit not only the degree of methylation of VEGF-C m6A but also the levels of VEGF-C, while METTL3-overexpressing ADSCs show the opposite effect. Further analysis indicated that IGF2BP2 binds to VEGF-C mRNA and that IGF2BP2 knockdown inhibits the levels of VEGF-C in ADSCs, indicating that ADSCs regulate VEGF-C expression via the METTL3/IGF2BP2-m6A pathway. This was the first study to clarify the specific mechanism by which ADSCs regulate VEGF-C. In addition, in vivo functional studies have shown that due to the high expression of VEGFR3, VEGF-C and LYVE-1, ADSCs regulate VEGF-C-mediated lymphangiogenesis in DFU mice via the METTL3/IGF2BP2-m6A pathway, indicating that ADSCs may regulate wound healing in DFU mice via the METTL3/IGF2BP2-m6A pathway. These findings were consistent with previous studies (Alishekevitz et al. 2016; Saaristo et al. 2006). In summary, our study showed that METTL3 could affect the expression of VEGF-C by regulating the m6A methylation level of VEGF-C. We found for the first time that IGF2BP2 binds to VEGF-C mRNA and affects the stability of VEGF-C mRNA through the m6A pathway.

## Conclusion

In summary, we have discovered a novel link between ADSCs and wound repair in DFUs. By expanding the mechanisms by which ADSCs enhance VEGFR3-mediated lymphangiogenesis via METTL3-mediated VEGF-C m6A modification, the results presented here reinforce their roles as potential therapeutic targets in wound repair of DFU.

## Data Availability

The raw data supporting the conclusions of this manuscript will be made available by the authors, without undue reservation, to any qualified researcher.

## Abbreviations

ADSCs: Adipose-derived mesenchymal stem cells
METTL3: Methyltransferase like 3
DFU: Diabetic foot ulcer
VEGF-C: Vascular endothelial growth factor C
VEGFR3: Vascular endothelial growth factor receptor 3
IGF2BP2: Insulin-like growth factor 2 binding protein 2
LECs: Lymphatic endothelial cells
AURKA: Aurora A kinase
RTKs: Receptor tyrosine kinases
m^6^A: N6-methyladenosine
